# Genomic signatures of introgression between commercial and native bumblebees, *Bombus terrestris*, in western Iberian Peninsula—Implications for conservation and trade regulation

**DOI:** 10.1111/eva.12732

**Published:** 2019-01-11

**Authors:** Sofia G. Seabra, Sara E. Silva, Vera L. Nunes, Vitor C. Sousa, Joana Martins, Eduardo Marabuto, Ana S. B. Rodrigues, Francisco Pina‐Martins, Telma G. Laurentino, Maria Teresa Rebelo, Elisabete Figueiredo, Octávio S. Paulo

**Affiliations:** ^1^ Centre for Ecology, Evolution and Environmental Changes (cE3c), Departamento de Biologia Animal, Faculdade de Ciências Universidade de Lisboa Lisboa Portugal; ^2^ Linking Landscape, Environment, Agriculture and Food (LEAF), Instituto Superior de Agronomia Universidade de Lisboa Lisboa Portugal; ^3^ Centre for Ecology, Evolution and Environmental Changes (cE3c), Natural History and Systematics (NHS) Research Group /MUHNAC ‐ Museu Nacional de História Natural e da Ciência Universidade de Lisboa Lisboa Portugal; ^4^ Zoological Institute University of Basel Basel Switzerland; ^5^ Centre for Environmental and Marine Studies (CESAM), Departamento de Biologia Animal, Faculdade de Ciências Universidade de Lisboa Lisboa Portugal; ^6^ Present address: SAPEC Agro Lisboa Portugal

**Keywords:** *Bombus terrestris*, bumblebees, cytochrome c oxidase subunit I (COX1), genome‐wide markers, introgression, pollination services, RAD sequencing

## Abstract

Human‐mediated introductions of species may have profound impacts on native ecosystems. One potential impact with largely unforeseen consequences is the potential admixture of introduced with autochthonous species through hybridization. Throughout the world, bumblebees have been deliberately introduced for crop pollination with known negative impacts on native pollinators. Given the likely allochthonous origin of commercial bumblebees used in Portugal (subspecies *Bombus terrestris terrestris* and *B. t. dalmatinus*), our aim was to assess their putative introgression with the native Iberian subspecies *B. terrestris lusitanicus. *We analysed one mitochondrial gene, cytochrome c oxidase subunit I (COX1) and genomic data involving thousands of genome‐wide restriction‐site‐associated DNA markers (RAD‐seq). In the mitochondrial COX1 analyses, we detected one relatively common haplotype in commercial bumblebees, also present in wild samples collected nearby the greenhouses where the commercial hives are used. In the RAD‐seq analysis, we found a clear genetic differentiation between native and commercial lineages. Furthermore, we detected candidate hybrids in the wild, as well as putatively escaped commercial bumblebees, some of which being potentially fertile males. Although we cannot assess directly the fitness effects of introgressed alleles, there is a risk of maladaptive allele introgression to the local bumblebee subspecies, which can negatively impact autochthon populations. One immediate recommendation to farmers is for the proper disposal of hive boxes, after their use in greenhouses, so as to minimize the risk of escapees contaminating native populations. On the other hand, the feasibility of using local subspecies *B. t. lusitanicus*, preferably with local production, should be evaluated.

## 1 INTRODUCTION

Agricultural practices may have profound impacts on native ecosystems, namely with the introduction of non‐native species (Goulson, 2003) which may become invasive, competing for resources or introducing diseases. These can also affect ecosystem interactions, such as plant–pollinator relationships (Matsumura, Yokoyama, & Washitani, 2004) or disrupt the genetic make‐up of local populations through hybridization with native species, with unforeseen consequences on the fitness of local populations (Ellstrand & Rieseberg, 2016; Gompert & Buerkle, 2016; Twyford & Ennos, 2012). In some cases, there may be a fitness increase in hybrids (“heterosis,” or “hybrid vigor”), caused by overdominance, masking of deleterious recessive alleles or epistatic interactions (Edmands, 1999). In fact, reports of species becoming invasive after hybridization events are widely known, such as weeds (Ellstrand et al., 2010) and Africanized honeybees in the New World (Hall, 1990; Pinto, Rubink, Patton, Coulson, & Johnston, 2005; Rangel et al., 2016). There is also concern that human‐modified or engineered genes may escape into the wild through hybridization (Ellstrand, 2001). On the other hand, a fitness decrease shown by hybrids (“outbreeding depression,” or “hybrid breakdown”), due to disruption of locally co‐adapted gene complexes or of favourable epistatic interactions (Lynch, 1991), may lead to a population decline (Edmands, 1999).

Bee species have been deliberately introduced in several parts of the world for pollination services in agriculture (Russo, 2016). They provide economic benefits by reducing costs in mechanical pollination, increasing fruit quality and yields (Velthuis & van Doorn, 2006) and decreasing the use of plant growth regulators. Nevertheless, the negative impact of these introduced species on wild pollinators has been reported before and may be one of the multiple causes for a global pollinator decline, endangering biodiversity and crop productivity (Goulson, Nicholls, Botias, & Rotheray, 2015).

One of the most widely used pollinator species is the buff‐tailed bumblebee, *Bombus terrestris* (Linnaeus, 1758) (Hymenoptera, Apidae), native from the West Palaearctic. It began to be artificially reared by commercial companies for greenhouse crop pollination, particularly tomatoes, in the 1980s (Goulson, 2010; Ings, Raine, & Chittka, 2005). Several traits have made *B. terrestris* highly suitable for commercial breeding: generalist feeding, efficient foraging, large colonies and flexible phenology (Dafni, Kevan, Gross, & Goka, 2010; Ings, Schikora, & Chittka, 2005; Velthuis & van Doorn, 2006). The potential for *B. terrestris* to behave as an invasive species results from its high dispersal and reproductive abilities, generalist foraging and flexible nesting habits, its thermoregulatory metabolism that allows it to be active at low temperatures (even during winter in some countries), its ability to compete with other bees for nest sites and flower resources (Dafni et al., 2010; Goulson, 2003, 2010 ; Ings, Ward, & Chittka, 2006) and to spread parasites and pathogens (Fürst, McMahon, Osborne, Paxton, & Brown, 2014; Goka, Okabe, Yoneda, & Niwa, 2001; Goulson & Hughes, 2015; Graystock, Goulson, & Hughes, 2014).

An additional threat posed by the use of *B. terrestris* comes from potential introgressive hybridization between commercial and native bumblebees. Commercial hives are provided with one queen, workers, a brood (eggs, larvae and pupae) and sugar solution for nectar supply. Workers are non‐reproductive, and only these are supposed to leave the nest to forage for nectar and pollen. However, later in the season, males and fertile females can be produced, and thus, hybridization with native species/subspecies may potentially occur. Although in the early years, the commercial rearing used several subspecies (including *B. t. lusitanicus*, *B. t. sassaricus* and *B. t. xanthopus*; Rasmont & Coppée, 2008), most commercial bumblebees used nowadays across Europe probably originate from stocks collected in northern Europe (subspecies *B. terrestris terrestris*) or the southeastern region in Greece and Turkey (subspecies *B. terrestris dalmatinus) *(Goulson, 2010; Lecocq, Coppée, et al., 2016; Lecocq, Rasmont, Harpke, & Schweiger, 2016; Rasmont & Coppée, 2008; Velthuis & van Doorn, 2006).

The nine *B. terrestris* subspecies distributed across the Mediterranean differ in external morphology (particularly colour pattern), physiological traits, resistance to parasites, behaviour and phenology (Rasmont & Coppée, 2008). Studies with mitochondrial and microsatellite variation have shown clear differences for some island subspecies, but found no differentiation among the mainland ones (Estoup, Solignac, Cornuet, Goudet, & Scholl, 1996; Widmer, Schmid‐Hempel, Estoup, & Scholls, 1998). Hybrids between different subspecies of *B. terrestris* are not difficult to obtain in captivity (Ings, Schikora, et al., 2005; Velthuis & van Doorn, 2006). Within its natural distribution range, the trade of the different subspecies of *B. terrestris* for crop pollination has no importation restrictions, with some exceptions, such as to the Canary Islands, Israel, Norway, Turkey and the UK, where only local subspecies are used (Lecocq, Coppée, et al., 2016; Moreira, Horgan, Murray, & Kakouli‐Duarte, 2015; Velthuis & van Doorn, 2006).

Genetic studies on detection of introgression between commercial and native *Bombus* populations are still scarce, and its magnitude and impact remain elusive. For instance, Kraus et al. (2011) in Poland used four microsatellite loci and found that the percentage of introgression from greenhouse commercial populations into wild ones was significantly higher in areas adjacent to greenhouse in comparison with more distant populations (>30 km). These results suggest that greenhouse commercial bumblebees introgress genetic material into the native conspecifics. In Moreira et al. (2015), the authors detected the most common mitochondrial COX1 haplotype from European continental populations in a few locations in Ireland, where it is mostly absent. One possible explanation for its presence in Ireland is once more, introgression from commercially bred populations. Furthermore, using eight microsatellites, they found limited genetic differentiation between commercial and some wild populations from Britain and continental Europe, suggesting that some wild samples could actually be commercial escapees and/or result from hybridization. A recent study, by Cejas, Ornosa, Muñoz, and De la Rúa (2018), reported potential hybrids between *B. t. terrestris* and *B. t. lusitanicus* in southern Spain (Sierra Nevada). Despite the uncertainty of morphological identification of these subspecies, these potential hybrids showed morphological characters of one subspecies and mitochondrial 16S haplotype of the other. Using microsatellites, Suni, Scott, Averill, and Whiteley (2017) did not detect widespread introgression between commercial and wild *Bombus impatiens* Cresson, 1863, in North America, despite some individuals collected in the wild showing >75% of genotype assignment to commercial stocks.

However, the reduced number of molecular markers used in these studies hindered a powerful inference of the magnitude and spatial distribution of introgression. Genome‐wide analyses of a large number of single nucleotide polymorphisms (SNPs) provide high resolution to detect and characterize introgression patterns (Muñoz et al., 2017, 2015 ; Payseur & Rieseberg, 2016; Pinto et al., 2014; Twyford & Ennos, 2012). Restriction‐site‐associated DNA sequencing (RAD‐seq) is one of the techniques available to efficiently identify and genotype thousands of SNPs across the genome for a large number of samples (Baird et al., 2008; Davey & Blaxter, 2010). It has been proven useful for the detection of introgression where traditional markers have failed (e.g., Eaton & Ree, 2013).

In the present study, we aimed to assess whether commercial bumblebee stocks (likely from subspecies *B. t. terrestris *and *B. t. dalmatinus*) are hybridizing with the local subspecies of the most western area of the species distribution, *B. t. lusitanicus*. We address this question by applying RAD sequencing analyses to estimate genetic ancestry of individuals and to test for signatures of introgressive hybridization (Gompert & Buerkle, 2016; Payseur & Rieseberg, 2016; Seehausen et al., 2014; Sousa & Hey, 2013; Twyford & Ennos, 2012). Given our sampling scheme and that the introduction of commercial bumblebees started no more than 30 years, we expect any potential hybridization events to be relatively recent.

Assessing whether introduced and native bumblebees are introgressing is essential not only to define conservation management plans but also to help shape regulations for bumblebee trading in order to diminish the risk of introgression of maladaptive alleles into local populations. This knowledge can aid further developments by companies commercializing bumblebees, for instance for local production of autochthonous bumblebees. This information is also important for farmers, who may use optimal pollination strategies and implement measures to diminish the risk of genetic contamination. In sum, this information is important both for biodiversity conservation and for agricultural productivity.

## 2 MATERIAL AND METHODS

### 2.1 Bumblebee sampling

**Figure 1:**
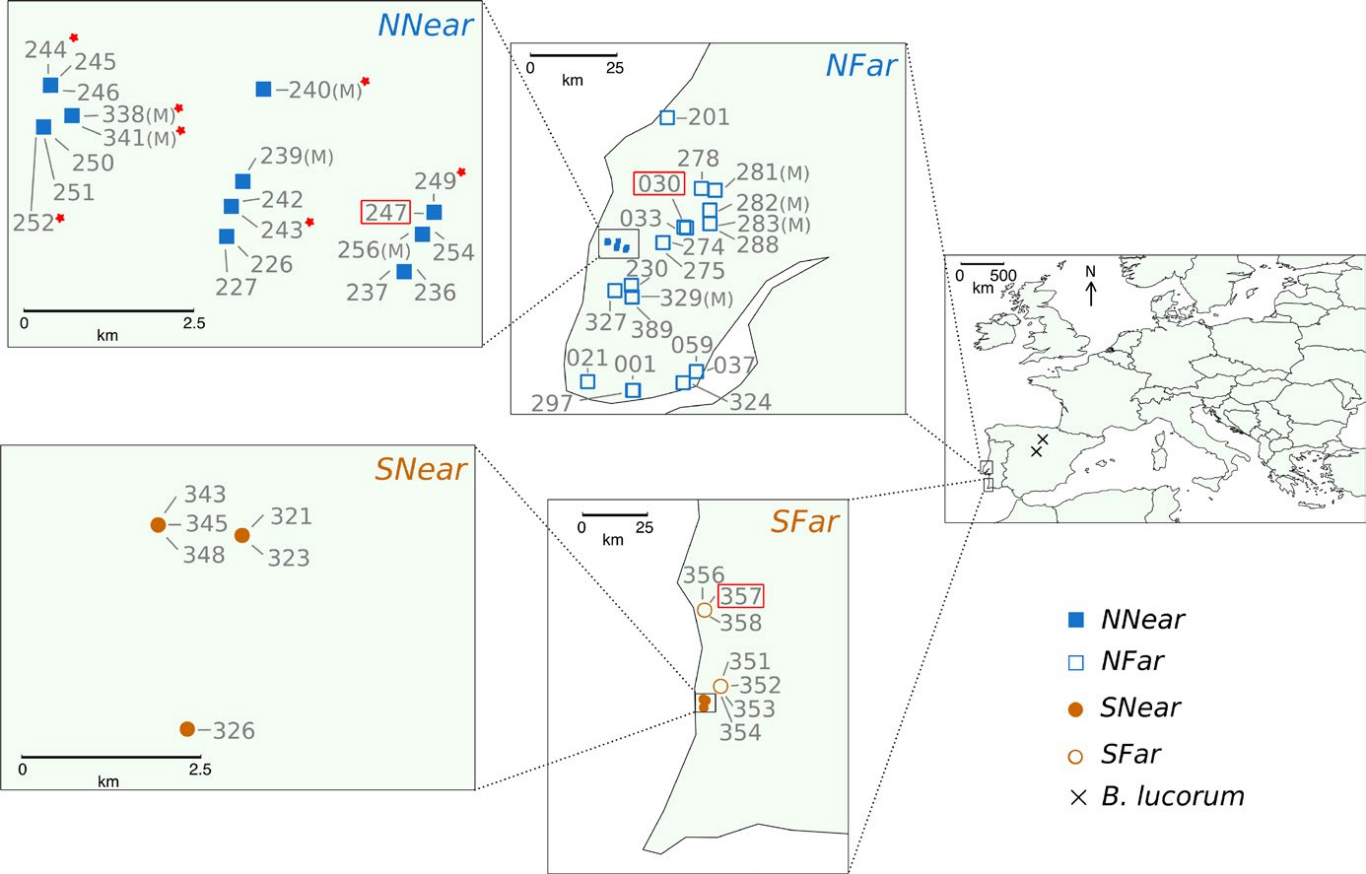
Sampling locations in the western Iberian Peninsula of each wild bumblebee used in this study. Individuals from the northern location are shown in blue squares and from the southern location in orange circles. *NNear*—northern location near greenhouses (blue filled squares); *NFar*—northern location far from greenhouses (blue empty squares); *SNear*—southern location near greenhouses (orange filled squares); *SFar*—southern location far from greenhouses (orange empty squares); *B. lucorum*—outgroup species (black crosses). Males (M), potential escapees (red stars) and potential hybrids (red rectangles) found in our study are also indicated

For the analysis of introgression between commercial and native *Bombus terrestris *in western Iberian Peninsula, we sampled both groups to characterize their genetic differentiation and the levels of admixture. We sampled 11 individuals from commercial hives and 53 from the wild. Commercial hives (*CH*) were collected at the exit of hives of five different trademarks used in Portugal (Supporting information Table S1). The wild samples were caught from two different areas in Portugal where commercial bumblebees are used for crop pollination (Figure 1; Supporting information Table S1), and where, due to historical usage of commercials in greenhouses in these two regions, it would be more likely to find hybrids between commercial and native bumblebees. The two regions were as follows: a) the northern region in our study (*N*), located in the “Oeste,” where the earliest use of commercial bumblebees in greenhouses in Portugal is recorded (since the early 1990's), mainly for tomato pollination, (Nunes, 1998); and b) the southern region in our study (*S*), in the “Sudoeste Alentejano” where their use is more recent (since the 2000's) for berry pollination (P. Brás de Oliveira, personal communication). Since these are geographically distinct regions and the effect of the difference in time since commercial use is unknown, we present the data separately for the two regions.

To account for the effect of distance from greenhouses, we divided the sampling points into two groups: “near” and “far” from greenhouses. *B. terrestris* has a reported foraging range of 1.5–2 km (Walther‐Hellwig & Frankl, 2000), with males being able to disperse at least 9.9 km (Kraus, Wolf, & Moritz, 2009). Queen dispersal in this species is unknown, but estimates for *B. pascuorum* and *B. lapidarius* indicate dispersal distances of at least 3 and 5 km, respectively. We considered the “near” group to be less than 5 km and the “far” group more than 5 km away from greenhouses. To avoid the confounding effects of isolation by distance in the analyses to detect introgression, we did not consider distances longer than 50 km, ensuring that there was no historical differentiation between the “near” and “far” groups. In the northern region (*N*), the near group (*NNear*) was considered to include samples collected less than 5 km from greenhouses (Supporting information Figure S1A) and the far group (*NFar*) from 10 to 50 km. In the southern group (*S*), the “near” group (*SNear*) includes samples collected less than 2 km from greenhouses (Supporting information Figure S1B) and the “far” group (*SFar*) from 6 to 36 km.

Sampling involved direct capture of individuals with a collection tube or using an entomological net. Most individuals were foraging female workers, but we also captured 10 males and 3 queens (Supporting information Table S1). Specimens were taken to the laboratory and frozen at −20ºC (dry or in absolute ethanol) for DNA preservation. Additionally, we sampled in Spain two specimens of *Bombus lucorum *(Linnaeus, 1761), a closely related species here used as outgroup (Supporting information Table S1).

### 2.2 DNA extraction and sequencing

Genomic DNA was extracted from 3 to 4 legs (fore and mid legs), the head and a portion of thorax of each individual using DNeasy Blood & Tissue extraction kit (Qiagen), following manufacturer's instructions. Tissues were finely cut with a scalpel before lysis. The lysate was centrifuged for 1 min at 13,000 g for precipitation of chitin residues, and the supernatant was transferred to the silica column of the kit.

Mitochondrial gene cytochrome c oxidase subunit I (COX1) was amplified using primers LEP‐F and LEP‐R (Hajibabaei, Janzen, Burns, Hallwachs, & Hebert, 2006), yielding a fragment of 620 bp. PCR volume of 15 µl contained 1× buffer (Promega), 1 mM of MgCl_2_, 0.1 mM of dNTPs, 0.4 µM of each primer and 0.04 U of *GoTaq Flexi* DNA polymerase (Promega). Polymerase chain reaction (PCR) conditions were as follows: 94°C for 1 min, 5 cycles of 94°C for 30 s, 45°C for 1 min and 72°C for 1 min, followed by 30 cycles of 94°C for 1 min, 50°C for 1.5 min and 72°C for 1 min, and a final extension of 5 min at 72°C. PCR products were purified with SureClean (Bioline), and Sanger sequencing of the forward sequence was done on an ABI3730XL, at Macrogen Europe. DNA sequences were checked and edited with Sequencher version 4.0.5 (Gene Codes Corporation).

RAD‐seq libraries were prepared following a protocol adapted from Etter, Preston, Bassham, Cresko, and Johnson (2011), available at https://www.wiki.ed.ac.uk/display/RADSequencing/Home. A total of 300 ng of genomic DNA of each of the 66 individuals was digested with restriction enzyme *PstI‐HF* (New England Biolabs) followed by ligation to 100 pmol of Barcoded P1‐modified Illumina Adapter. Individually barcoded samples were multiplexed and sheared targeting a 500 bp average size in a Bioruptor (Diagenode) using 10 cycles of 30 s and purified using the MinElute PCR Purification Kit (Qiagen), followed by an extra purification step with Agencourt AMPure XP (Beckman Coulter) magnetic beads. Fragments between 300 and 600 bp in length were selected by gel extraction and purified using the MinElute Gel Purification Kit (Qiagen). After end‐repair and 3’‐dA overhang addition, libraries were purified using the same kit. After P2 adapter ligation, libraries were purified using Agencourt AMPure XP magnetic beads. A final library amplification was done by PCR as follows: an initial denaturation step at 98°C for 30 s, followed by 18 cycles of one denaturation step at 98°C for 10 s, annealing at 65°C for 30 s, extension at 72°C for 30 s and a final 5 min extension step. PCR‐enriched libraries were purified using Agencourt AMPure XP magnetic beads. The DNA concentration of each library was quantified in Qubit 2.0 (Invitrogen), using Qubit dsDNA HS Assay kit, and the same proportional representation of each individual was used in the final volume. Paired‐end sequencing was done in Illumina HiSeq 2000/2005 at Edinburgh Genomics, Ashworth Laboratories (https://genomics.ed.ac.uk/). The 66 individuals were ran together with other 42 samples for another study (S. E. Silva, unpublished), over two lanes.

### 2.3 Mitochondrial DNA analysis

Chromatogram peak calling was done in Sequencher 4.05 (Gene Codes Corporation). Sequence alignment was performed in Mafft version 7.205 (Katoh & Standley, 2013) using default settings and checked for accuracy using BioEdit version 7.2.5 (Hall, 1999). Alignment files were converted to NEXUS format using Concatenator version 1.1.0 (Pina‐Martins & Paulo, 2008). A median‐joining haplotype network was constructed using Network 4.5.1.0 (Bandelt, Forster, & Rohl, 1999; fluxus‐engineering.com). The designation of haplotypes followed S. E. Silva (unpublished) that included a wider sampling of the Iberian Peninsula and a larger number of haplotypes.

### 2.4 RAD sequencing analysis

RAD sequence reads were processed using *process_radtags* available in Stacks version 1.45 (Catchen, Hohenlohe, Bassham, Amores, & Cresko, 2013) to filter for quality and to demultiplex based on individual barcodes. The reads of each individual were aligned to the assembled reference genome of *B. terrestris* (https://www.ncbi.nlm.nih.gov/assembly/GCA_000214255.1) using Bowtie2 version 2.1.0 (Langmead & Salzberg, 2012) with the ‐‐*sensitive* option and with the last three bases from the 3’ end of each read trimmed before alignment (‐‐trim3 3). The resulting BAM files were filtered to exclude low‐quality alignments (‐q 20) and unmapped reads (‐F 0x0004) using SAMtools version 0.1.19 (Li et al., 2009). Around 60% of the reads retained from *process_radtags* were successfully mapped against the reference and properly paired (Supplementary Table S1). Stacks were then used to obtain RAD loci and SNPs, by running *pstacks* considering a minimum depth of coverage of 10 (‐m 10), followed by *cstacks* and *sstacks*, using base matching on alignment position (‐g).

Three data sets were produced as follows: TOTAL, FEMALES and NO_OUTGROUP. From the initial TOTAL data set, we excluded males to produce data set FEMALES. This was done because males are haploid and most inference methods are tailored to diploid data. From this last data set, the two individuals of *B. lucorum* were excluded, producing data set NO_OUTGROUP, for the intra‐specific analyses.

All three data sets were obtained using *populations *(Stacks software) with the parameters: minimum minor allele frequency required to process a site (‐‐min_maf) of 0.05; minimum number of populations a locus must be present in to process a locus (‐p) of 6 or 5 (with or without the outgroup, respectively); minimum percentage of individuals in a population required to process a locus for that population (‐r) of 50; and only one SNP from each RAD tag retained (‐‐write_random_snp). We additionally excluded repeated sites (mostly from overlapped paired‐end reads from different RAD tags) and SNPs with more than 25% missing individuals and with mean coverage higher than 200×, using VCFtools. To obtain the initial data set TOTAL, we excluded individuals that had a number of reads lower than the 15% quantile.

We performed principal component analysis (PCA) on the three data sets using the R package SNPRelate version 1.12.0 (Zheng et al., 2012). Based on PCA results, we defined groups of samples.

For the data set TOTAL, we obtained observed and expected heterozygosity, as well as F_IS_ using VCFtools version 0.1.14. Differentiation between groups was obtained from pairwise F_ST_ estimated using Arlequin 3.5.1.3. Permutation tests with 10,000 repetitions were applied to obtain the significance of *F*_ST_.

### 2.5 Admixture analyses

To obtain ancestry proportions for the data set FEMALES, we used the Bayesian model‐based clustering approach available in Structure v. 2.3.4 (Falush, Stephens, & Pritchard, 2003; Pritchard, Stephens, & Donnelly, 2000), using the admixture model and assuming correlated allele frequencies among populations. We tested the number of clusters (K) from 1 to 6, running 10 replicates of each, with 500,000 steps of burn‐in and 1,000,000 MCMC steps after burn‐in. All other parameters were set to default. The program Structure_threader version 1.2.2 (Pina‐Martins, Silva, Fino, & Paulo, 2017) was used to parallelize the runs and find the K best explaining the data (Earl & vonHoldt, 2012; Evanno, Regnaut, & Goudet, 2005). The 10 replicate runs of Structure for each K were permuted to align the clusters across runs using CLUMPP version 1.1.2 (Jakobsson & Rosenberg, 2007) to obtain the optimal alignment of ancestry proportions.

To obtain estimates of genome‐wide admixture (hybrid index) for each individual in the data set NO_OUTGROUP, we applied the Bayesian genomic cline model, based on allele frequency differences in parental populations, implemented in the R package Introgress v. 1.2.3 (Gompert & Buerkle, 2009, 2010 ). In this approach, we need to define a priori the parental populations. One parental population corresponded to the *CH* samples and the other to the *NFar* samples, excluding the potential hybrid detected in the PCA and Structure. For the calculation of parental allele frequencies, we re‐sampled without replacement five individuals of each parental population and repeated this 10 times. Mean hybrid indices were then calculated based on this variation in allele frequencies.

To detect potential hybrids, we used the Bayesian clustering method implemented in NewHybrids v. 1.1 beta (Anderson & Thompson, 2002; https://github.com/eriqande/newhybrids). This method estimates the posterior probability for each individual belonging to distinct genotype categories, either parentals (P1 or P2) or hybrids (F1, F2, backcross with P1 or backcross with P2). We used the most differentiated SNPs between *CH* and *NFar* (excluding from this last group the potential hybrid detected with PCA and Structure that may not be a true parental). This data set (MOST_DIFFERENTIATED) was obtained from the NO_OUTGROUP data set and represents the 1% of SNPs with highest *F*_ST_ (higher than 0.39). We did not assign a priori any individual to any of the categories, and we ran 1,000,000 iterations with a burn‐in of 10,000.

### 2.6 *D*‐statistics

To test for genome‐wide evidence of introgression between commercial and native populations accounting for incomplete lineage sorting, we used the *D*‐statistics, also known as “ABBA/BABA test” (Durand, Patterson, Reich, & Slatkin, 2011; Patterson et al., 2012). Based on the number of alleles shared among populations, the values of the *D*‐statistic allow to distinguish introgression (allele sharing due to gene flow) from incomplete lineage sorting. Given a fixed population tree topology of the type (((P1,P2),P3),O), in the absence of gene flow, the number of sites where populations P2 and P3 share the same allele (ABBA pattern) is expected to be the same as the number of sites where populations P1 and P3 share the same allele (BABA pattern), resulting in an expected *D*‐statistic of zero. However, gene flow between P3 and P2 leads to an excess of sites with the ABBA allele sharing pattern, resulting in significantly positive *D*‐statistic values. In our case, populations P1 and P2 correspond to native populations, P3 corresponds to the *CH* samples, and the outgroup (O) corresponds to *B. lucorum*. We tested whether the allele frequencies across samples indicated gene flow (significant positive and/or negative *D*‐statistics). For each target population (P2), we performed the *D*‐statistic test against the three other native populations as the P1 (e.g., for *NNear* as the target population, we computed three *D*‐statistics with P1 either as *NFar*, *SNear* or *SFar*). Given that our samples might comprise a mixture of individuals with different degrees of introgression, we also tested for evidence of gene flow between *CH* (P3) and a target population (P2) at the individual level. We computed the *D*‐statistic for each individual of the target population (P2) conditional on the allele frequencies of P1, P3 and the outgroup. Significant positive *D*‐statistics would indicate gene flow between *CH* and the lineage of the target individual. The *D*‐statistics were computed based on sample allele frequencies (Durand et al., 2011) accounting for missing data. We used the FEMALES data set obtained from *populations *(Stacks software)*.* To maximize the number of sites, we did not filter for missing data per population, and instead computed the allele frequencies accounting for missing data (i.e., accounting for differences in the number of genotyped individuals at a given site in a given sample), which resulted in data set D‐STAT. To account for the presence of linked sites, significance of the *D*‐statistics was assessed with a block‐jackknife approach (Busing, Meijer, & Leeden, 1999), dividing the data set into 50 blocks, following Martin et al. (2013).

### 2.7 Direction of gene flow

To investigate the relationship between commercial and native populations, we inferred the population tree that better explains the allele covariance matrix across populations using the diffusion approximation model implemented in TreeMix v1.13 (Pickrell & Pritchard, 2012). We inferred the population tree for models without migration and with a variable number of migration edges (single pulses of migration). TreeMix estimates both the position of migration edges in the tree and the direction of gene flow. Thus, we inferred populations that exchanged migrants and the direction of introgression based on the estimated migration edges. We tested the fit of models with 0–3 migration edges. For each model, we performed 10 independent runs, selecting the one with the lowest sum of square residuals (i.e., minimizing the difference between the observed and expected covariance matrix of allele frequencies). We used the same data set as for the *D*‐statistic analysis but discarding the outgroup (*B. lucorum*) and sites with missing data in the remaining five populations, which resulted in data set D‐STAT_NO_OUTGROUP.

Input files were converted using PGDSpider 2.0.3.0 (Lischer & Excoffier, 2012) or custom python scripts (https://doi.org/10.6084/m9.figshare.5962522.v1). Statistical analyses were done using R version 3.4.0 (R Development Core Team, 2008).

## 3 RESULTS

**Figure 2:**
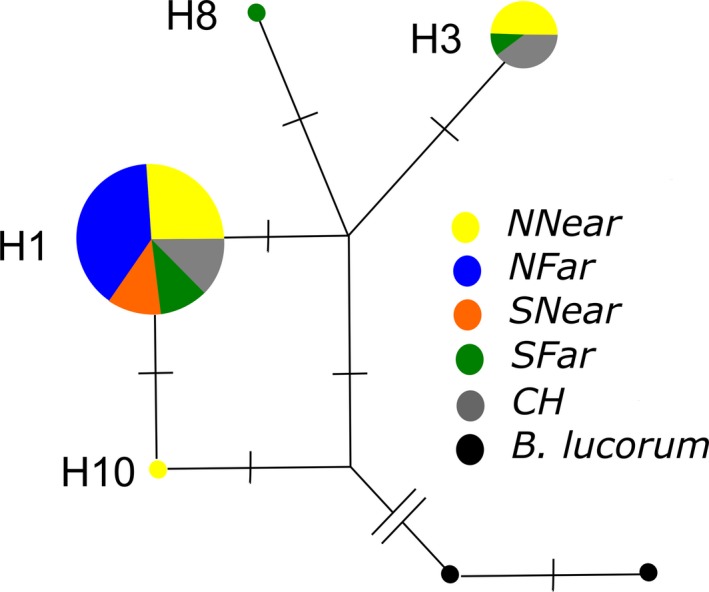
Haplotype median‐joining network of mitochondrial COX1 sequences of *Bombus terrestris* (collected at commercial hives and in the wild near or far from greenhouses in two regions in Portugal, North and South). Haplotypes of *Bombus lucorum*, the outgroup species, are also included. The size of the circles is proportional to the number of haplotypes, and the number of base substitutions between haplotypes is indicated by perpendicular line segments, except for the line separating *Bombus terrestris* from *Bombus lucorum* which has 45 substitutions

Among the 64 *B. terrestris *specimens genotyped for mtDNA COX1 gene, we recovered their clustering into four haplotypes with one to four base substitutions between them (Figure 2; Supporting information Table S1; GenBank Accession numbers MH018608—MH018673). Haplotype H1 was the most common one in every group of samples: *CH*, *NNear*, *NFar*, *SNear* and *SFar*. Haplotype H3 was found in four out of 11 *CH* individuals. It was also present in five out of 20 individuals of *NNear* and in one out of three individuals of *SFar*. *B. lucorum* haplotypes differed from *B. terrestris* by 45–47 substitutions.

We obtained an average of 7.6 million paired‐end reads (of 120 bp) per individual, and after filtering with *process_radtags*, 7.3 million of those were retained (Supplementary Table S1). Nine individuals had low number of markers (less than 2.8 million retained reads; Supplementary Table S1) and were thus excluded from further analysis. Individual BTL_146 also had low number of reads (1.47 million retained reads), but was not excluded because we had a limited sample of two *B. lucorum* individuals. The TOTAL data set comprises 57 individuals and 27,898 SNPs, with a mean coverage of 56.6 x per site and per individual.

**Figure 3:**
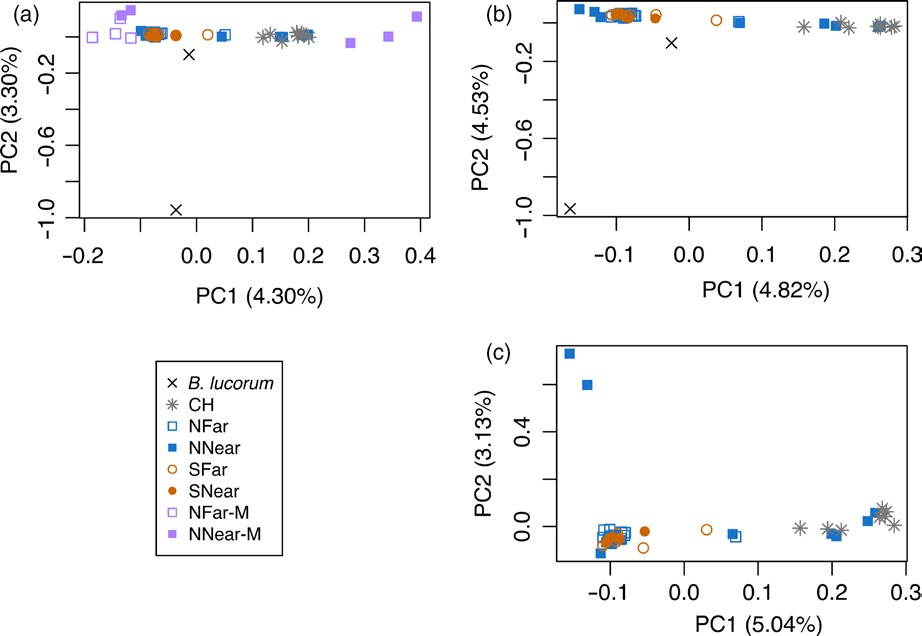
Principal components analysis (PCA) of data sets TOTAL (a), FEMALES (b) and NO_OUTGROUP (c). *NNear*—northern location near greenhouses; *NFar*—northern location far from greenhouses; *SNear*—southern location near greenhouses; *SFar*—southern location far from greenhouses; *CH*—Commercial hives; *B. lucorum*—outgroup species

Principal components analysis (PCA) for the TOTAL data set showed *B. terrestris* segregating into several groups in principal component 1 (PC1) (Figure 3a), while in PC2, *B. lucorum* separates from *B. terrestris*. One of the two individuals of *B. lucorum* (BTL_146) was much closer to *B. terrestris* than the other, which probably derives from its high level of missing data (60.8% of missing SNPs). This data set includes nine *B. terrestris* males which, unlike females, are haploid. Two groups of highly differentiated males were found, falling into the two extremes of PC1 (Figure 3a). The three males falling into the positive extreme of PC1 (BTL_240, BT_338 and BT_341), belonging to *NNear*, cluster nearer to the commercial than the wild samples. Two of these have been collected within metres from a greenhouse and had been considered possible escapees (*PE‐M*). One of them (BT_338) carried the mitochondrial haplotype H3. The remaining males fell into the negative extreme of PC1, closer to wild‐caught than to commercial samples. These include two specimens from *NNear* (*NNear‐M*) and four from *NFar* (*NFar‐M*) (Figure 3a). Excluding the nine males from this data set, we obtained the data set FEMALES (48 individuals for 15,984 SNPs), and further excluding the outgroup, we obtained the data set NO_OUTGROUP (46 individuals for 17,681 SNPs). PCAs of these two data sets (Figure 3b,c, respectively) revealed that commercial *CH* samples formed a cluster with four females from *NNear* (BTL_243, BTL_244, BTL_249, BTL_252; Figure 3b,c). Three of these carried the mitochondrial haplotype H3. We considered these as possible commercial bumblebees escaped from the greenhouses (*PE‐F*). Another group in the other extreme of PC1 was formed by most of the wild samples. When excluding the outgroup, two samples from *NNear* (BTL_250 and BTL_251) formed a separate cluster in PC2 (Figure 3c). In the PC1, three samples fell in an intermediate position between commercial and wild samples (Figure 3b,c) and might represent potential hybrids (*Hyb*). One (BTL_247) was from *NNear* (carrying mitochondrial haplotype H3), another (BTL_030) from *NFar* (with mitochondrial haplotype H1) and the other (BTL_357) from *SFar* (also with mitochondrial haplotype H3).

The group of putatively escaped females (*PE‐F*) showed low differentiation from the commercial individuals (*CH*) (*F*_ST_ =0.006; Table 1) and higher and significant differentiation from the wild female groups (*NNear*, *NFar*, *SNear* and *SFar*; *F*_ST_ between 0.045 and 0.051; Table 1). *F*_ST_ values between the group of putative hybrids (*Hyb*) and commercial individuals (*CH*) (*F*_ST_ = 0.017) were similar to those found between *Hyb* and wild populations (*NNear*, *NFar*, *SNear* and *SFar*; *F*_ST_ between 0.014 and 0.021; Table 1). Differentiation between commercial and wild populations ranged from 0.041 to 0.046, while between pairs of wild populations were lower than 0.013 (Table 1). Ploidy influences the differentiation of individuals (Wragg et al., 2018), and thus, we did not compare male and female *F*_ST_. Commercial and wild females did not differ in mean diversity (expected heterozygosity) and *F*_IS_ (Table 1; He: *t* test, *t* = 1.1926, *df*=16.808, *p* = 0.2496; *F*_IS_: *t* test, *t *= −0.0838, *df *= 13.835, *p* = 0.934).

**Table 1:** Mean pairwise *F*_ST_ values between groups of individuals from data set TOTAL

	*CH*	*PE‐F*	*Hyb*	*NNear*	*NFar*	*SNear*	*SFar*	*NNear‐M*	*Nfar‐M*	*PE‐M*	*B. lucorum*
*N*	8	4	3	8	13	5	5	2	4	3	2
Ho	0.253	0.247	0.260	0.268	0.259	0.229	0.235	0.009	0.011	0.009	0.134
He	0.287	0.287	0.287	0.287	0.287	0.286	0.286	0.288	0.288	0.288	0.287
*F*_IS_	0.117	0.140	0.093	0.067	0.097	0.200	0.180	0.967	0.962	0.967	0.531
*CH*	0										
*PE‐F*	0.006	0									
*Hyb*	0.017	0.018	0								
*NNear*	0.046**	0.051*	0.021	0							
*NFar*	0.042***	0.045**	0.014*	0.006	0						
*SNear*	0.041**	0.049*	0.020*	0.007	0.004	0					
*SFar*	0.042**	0.046*	0.020*	0.012	0.006	0.009	0				
*NNear‐M*	0.153	0.175	0.164	0.135	0.130*	0.154	0.146	0			
*Nfar‐M*	0.103*	0.115	0.088	0.074*	0.067**	0.079*	0.081*	0.221	0		
*PE‐M*	0.095*	0.103	0.117	0.129*	0.124*	0.140*	0.136*	0.283	0.201	0	
*B. lucorum*	0.076	0.109	0.111	0.073	0.058	0.097	0.088	0.347	0.178	0.245	0

Note

Number of individuals (*N*), mean observed heterozygosity (Ho), mean expected heterozygosity (He) and mean *F*_IS_ for each group are shown at the top of the table.

Significance levels after FDR correction (Benjamini & Yekutieli, 2001) (n = 55):

* 0.01 < *p*<0.05;

** 0.001 < *p*<0.01;

*** *p* < 0.001.

**Figure 4:**
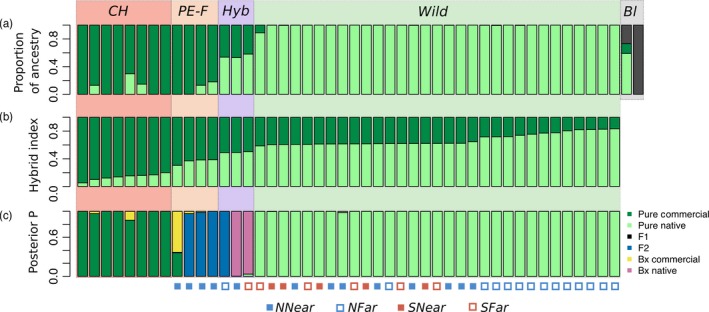
(a) Admixture proportions of each individual from *K* = 3, obtained in Structure for data set FEMALES; (b) genome‐wide admixture (hybrid index) for each individual, obtained in Introgress for data set NO_OUTGROUP; (c) posterior probability that each individual belongs to distinct genotype categories, either parentals (Pure Commercial or Pure Native) or hybrids (F1, F2, backcross with commercial or backcross with native), obtained in NewHybrids for data set MOST_DIFFERENTIATED. *CH*—commercial hives; *PE‐F—*possible escaped females; *Hyb*—potential hybrids; *Wild*—all other females collected in the wild; *Bl*—*Bombus lucorum*. Individuals are sorted according to hybrid index. Symbols in the bottom represent the groups where individuals come from, either *NNear, NFar, SNear* or *SFar*

Structure harvester revealed a most likely *K* of 3 (Figure 4a). *CH* individuals showed high ancestry proportions from group 2 (70.3%–100%), while most wild‐caught individuals had high ancestry proportion from group 1 (Figure 4a; Supporting information Table S1). The four possible female escapees from *NNear* in the PCA presented ancestry proportions similar to those from *CH* (82.1%–100% from group 2). The three potential hybrids detected in the PCA showed intermediate levels of ancestry (53.1%–58.1% from group 1). *B. lucorum* was assigned to group 3, although BTL_146 shared high ancestry with group 1, probably as a result of the high level of missing loci for this individual.

We estimated hybrid indices with Introgress for the data set NO_OUTGROUP. Among specimens considered as possible escapees from commercial hives, hybrid indices ranged from 0.3 to 0.4. Potential wild x commercial hybrids had hybrid indices of 0.5 (Figure 4b).

We used NewHybrids to further detect hybridization between wild and commercial bumblebees with the MOST_DIFFERENTIATED data set. We only considered SNPs showing the highest differentiation between *CH* samples and the group *NFar *(excluding all potential hybrids from this group), in a total of 177 SNPs. The analysis revealed a high posterior probability of three of the possible escaped individuals being an F2 and the other being a backcross with commercials (Figure 4c). Two of the potential hybrids have high posterior probability of resulting from a backcross with the wild *B. terrestris* and the other of being an F2.

**Figure 5:**
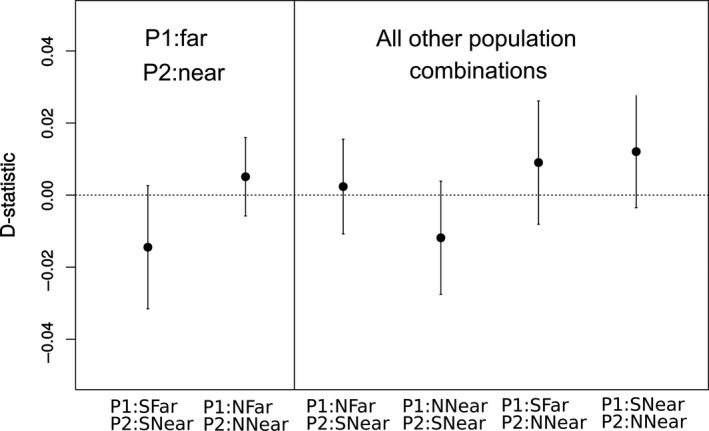
Test for gene flow with commercial using D‐statistic computed at the population level for all population combinations for P1 and P2 with P3: Commercial (*CH*) and P4: outgroup *B. locurum *(i.e., D(P1, P2, P3:*CH*, P4:outgroup)). No D‐statistic was significantly different from zero

**Figure 6:**
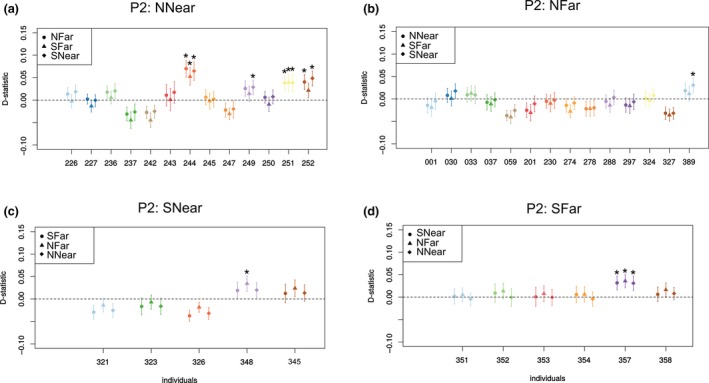
Test for gene flow between commercial and individuals from a target population (P2) using D‐statistic computed at the individual level for all population combinations of P1 and P2, fixing P3: Commercial (*CH*) and P4: outgroup *B. lucorum *(i.e., D(P1, P2, P3:*CH*, P4:outgroup)). (a) Target population (P2) *NNear*, (b) target population (P2) *NFar*, (c) target population (P2) *SNear*, (d) target population (P2) *SFar*. The D‐statistic for each individual of the target population was computed against the three alternative P1 populations (shown as different points). Asterisks indicate significant positive *D*‐statistics (*p* < 0.05)

The data set D‐STAT had a total of 42,928 SNPs. From this data set, we obtained the data set to compute the *D*‐statistic for each combination of populations by keeping only sites with data in at least one individual from each population. Hence, the number of SNPs for each *D*‐statistic computation ranged from 16,599 to 27,760. In agreement with introgression of *CH* into *NNear*, the *D*‐statistics computed at the population level suggest that *NNear* shares more alleles with *CH*, as indicated by positive values when *NNear* is the P2 and by negative values when *NNear* is the P1 (Figure 5). In contrast, results suggest that *SFar* shares more alleles with *CH* than *SNear* (Figure 5). Note, however, that these results were not significantly different from zero. We detected significant evidence of gene flow with *CH* when performing the test for each individual of the target population (P2) (Figure 6). We found significant positive *D*‐statistics for some individuals, indicating introgression from commercials (Figure 6; Supporting information Figure S2). For the target *NNear* (Figure 6a), we find evidence of introgression in three out of 13 females (BTL_244, BTL_251 and BTL_252; Figure 5). Two of these, BTL_244 and BTL_252, also considered as possible escapees by PCA and Structure, had hybrid indices (Introgress) of 0.31 and 0.37, respectively, and NewHybrids identified the first as a backcross with commercials and the second as an F2. Female BTL_251 was identified as a pure wild (Structure and NewHybrids) with a hybrid index of 0.65 (Introgress). For the target *SFar* (Figure 6d), female BTL_357, which was classified as potential hybrid by PCA, Structure, Introgress (0.50) and as a backcross with the wild population by NewHybrids, also showed a consistent positive *D*‐statistic. These results are consistent with the ones from gene flow tests at the individual level, when each individual of the target population (P2) is tested against each individual of P1 (Supporting information Figure S3). The *D*‐statistic evidences a relatively higher allele sharing between P2 and *CH*, which could result from gene flow in both directions. To investigate whether gene flow was from *CH* into P2 or from P2 into *CH,* we inferred the population tree that best fitted the allele covariance matrix. We used data set D‐STAT_NO_OUTGROUP, with 32,648 SNPs. Our estimates support a recent introgression of *CH* into *NNear* (TreeMix results in Supporting information Figure S4), which is in agreement with the PCA, Structure and *D*‐statistic results.

## 4 DISCUSSION

We applied genetic analyses to detect signatures of introgression between commercial and native *Bombus terrestris* in western Iberian Peninsula, where the native subspecies is *B. t. lusitanicus*. In the mitochondrial analysis, we found one COX1 haplotype (H3) to be relatively common in commercial bumblebees, which was also detected in the greenhouse areas investigated. This haplotype has not been found in any other area of the Iberian Peninsula so far, except for one specimen collected in southeastern Spain, in Murcia (S. E. Silva, unpublished), about 300 km from the area where Cejas et al. (2018) detected potential hybrids between these subspecies based on morphological and mitochondrial 16S data. In the south of Spain, commercial bumblebees, from the same companies trading in Portugal, are also used in greenhouses. Since such stocks include mostly subspecies *B. t. terrestris* and *B. t. dalmatinus* (Lecocq, Coppée, et al., 2016; Velthuis & van Doorn, 2006), it is possible that this haplotype is prevalent in one or both of these subspecies. However, it was not detected so far in individuals sampled across Europe (Moreira et al., 2015; S. E. Silva, unpublished).

Genome‐wide analyses with RAD sequencing allowed for a finer‐scale evaluation of both the genetic differentiation between commercial and native populations of *B. terrestris* but also enabled to find evidence of the occurrence of hybrids near the points of contact of these different gene pools. Three potential wild‐caught hybrids were detected by PCA and also by analyses carried out with Structure, Introgress and NewHybrids methods. Only one of them was detected with the *D*‐statistics, as expected given that *D*‐statistics has less power for cases of recent admixture and little differentiation among populations (Durand et al., 2011). Moreira et al. (2015) found that commercial bumblebees were differentiated from the majority of the wild populations from Ireland, having a high number of unique microsatellite alleles. Similarly to that study, we found that inbreeding was not higher in commercial colonies than in wild populations, contrarily to what would be expected for populations breeding in controlled laboratory conditions. This may be an effect of mixing several stocks for creating commercial breeds. To test this hypothesis and understand their relative contribution for the gene pool of commercial stocks, both subspecies putatively used for commercial breeding, *B. t. terrestris* and *B. t. dalmatinus*, should be genotyped. In agreement with Lecocq, Coppée, et al. (2016), our results stress the need for trade companies to provide information on the taxonomic identity or geographic origin of the strains used for an efficient trade regulation.

We found that four females and three males caught outside greenhouses likely represent escapees from commercial hives. The four females from *NNear* are even assigned as F2 hybrids or backcrosses with commercial individuals, and two of them showed significant positive *D*‐statistics, indicating introgression with commercial lineages. *D*‐statistics and TreeMix results, at the population level and even more at the individual level, reinforced the evidence for introgression between *CH* and *NNear*. However, obtaining precise individual estimates of the level and timing of admixture would require data from more individuals and more genomic markers, namely by using whole genome resequencing.

We found males next to one greenhouses in June and July (at the end of the first of two annual tomato crop seasons) that were genetically more similar to commercial samples. Since males may be fertile, their presence indicates an increased risk of introgression with wild bumblebees. We observed the substantial occurrence of males inside commercial hives after their usage in greenhouses (observations by S.G.S. and E.F.) and of considerable numbers of large females, possibly new queens (gynes), which was similar to what was reported by Ings et al. (2006). Interestingly, we found a new nest being built between the outer card box and the inner plastic box in one of the commercial hives left outside the greenhouse after usage. If these were commercial bumblebees, it may indicate that they are able to persist after their intended usage. If they were wild instead, it represents an increased risk of interbreeding between both gene pools. Finally, the finding of individuals with mixed ancestry (hybrid index close to 0.5 – Figure 4, Supporting information Table S1) is an indication that hybrid nests may be already establishing in the wild.

In this study, we analysed two geographical regions that differed by around ten years in the time since the use of commercial bumblebees. The limited sample size, especially in the southern region, does not allow a robust comparison of this effect, but the fact that we found potential hybrids in both regions and some far from the greenhouses may suggest that increased sampling will unveil a higher prevalence of hybrids than here detected.

We alert to the risk of maladaptive introgression resulting from the use of allochthonous commercial bumblebees. Locally adapted gene complexes may be disrupted through introgression of maladaptive alleles, with potential negative fitness consequences (Bolnick & Nosil., 2007; Roesti, 2018). Although we cannot assess directly the fitness effects of introgressed alleles, given that there is some divergence between commercial and native species (*F*_ST_ values between these groups larger than 0.041), it is likely that introgressed alleles from commercial individuals are less fit in the local habitat. The issue is of particular concern when commercial hives are used in open‐field crops (berries, pear orchards) or Mediterranean greenhouses, which generally have more open structures than other greenhouse types, and where contact with native individuals is much harder to avoid. One immediate recommendation we can make based on this study's results is that hive boxes which are no longer in use should not be left outside greenhouses, but rather properly disposed of by either freezing, or sealing in a closed box.

The use of bumblebees for pollination should not be discouraged since it is preferable than the alternative of using potentially environmental hazardous plant growth regulators. Furthermore, using bumblebees for pollination led to changes in pest management practices since farmers started to look for solutions which are safe for bumblebees, since these became a valuable asset. However, long‐term ecological impacts of using non‐native pollinators should be taken into account when trading and importation regulations are defined (Dafni et al., 2010; Velthuis & van Doorn, 2006; Winter & Adams, 2006). The viability of using native bumblebees for commercial purposes, preferably with local production, should be investigated and promoted. This might involve extra costs, and companies may not be willing to apply such changes without legislation. The establishment of a monitoring scheme of bumblebee populations to detect admixture could be integrated in a conservation programme including prevention of pathogen spillover from commercial into native bumblebees (Murray, Coffey, Kehoe, & Horgan, 2013) that would help to improve species distribution models for invasive risk assessment (Lecocq, Rasmont, et al., 2016). The genome‐wide molecular markers here obtained, particularly those SNPs showing highest differentiation between commercial and Iberian bumblebees, can be used to develop a SNP panel for easy genotyping.

Data for this study are available at: GenBank (accessions MHO18608—MHO18673) and Sequence Read Archive (accession SRP134176)

## CONFLICT OF INTEREST

None declared.

## AUTHOR CONTRIBUTIONS

SGS, VLN, MTR, EF and OSP performed delineation of the experiment; SES, VLN, JM, EM, ASBR, TGL, EF and OSP conducted sampling; VLN carried out the molecular laboratory work; SGS, SES, VLN and VCS conducted data analysis; SGS and SES wrote the manuscript; all authors performed discussion of results and revision of the manuscript.
