# Supplementary material for: Genomic signatures of introgression between commercial and native bumblebees, Bombus terrestris, in western Iberian Peninsula—Implications for conservation and trade regulation
Source: Evol Appl. 2019 Jan 11;12(4):679–91. doi: 10.1111/eva.12732 (PMC6439495; doi:10.1111/eva.12732)
Supplement: Supplementary file 1 [file EVA-12-679-s001.pdf]

Supplementary Material

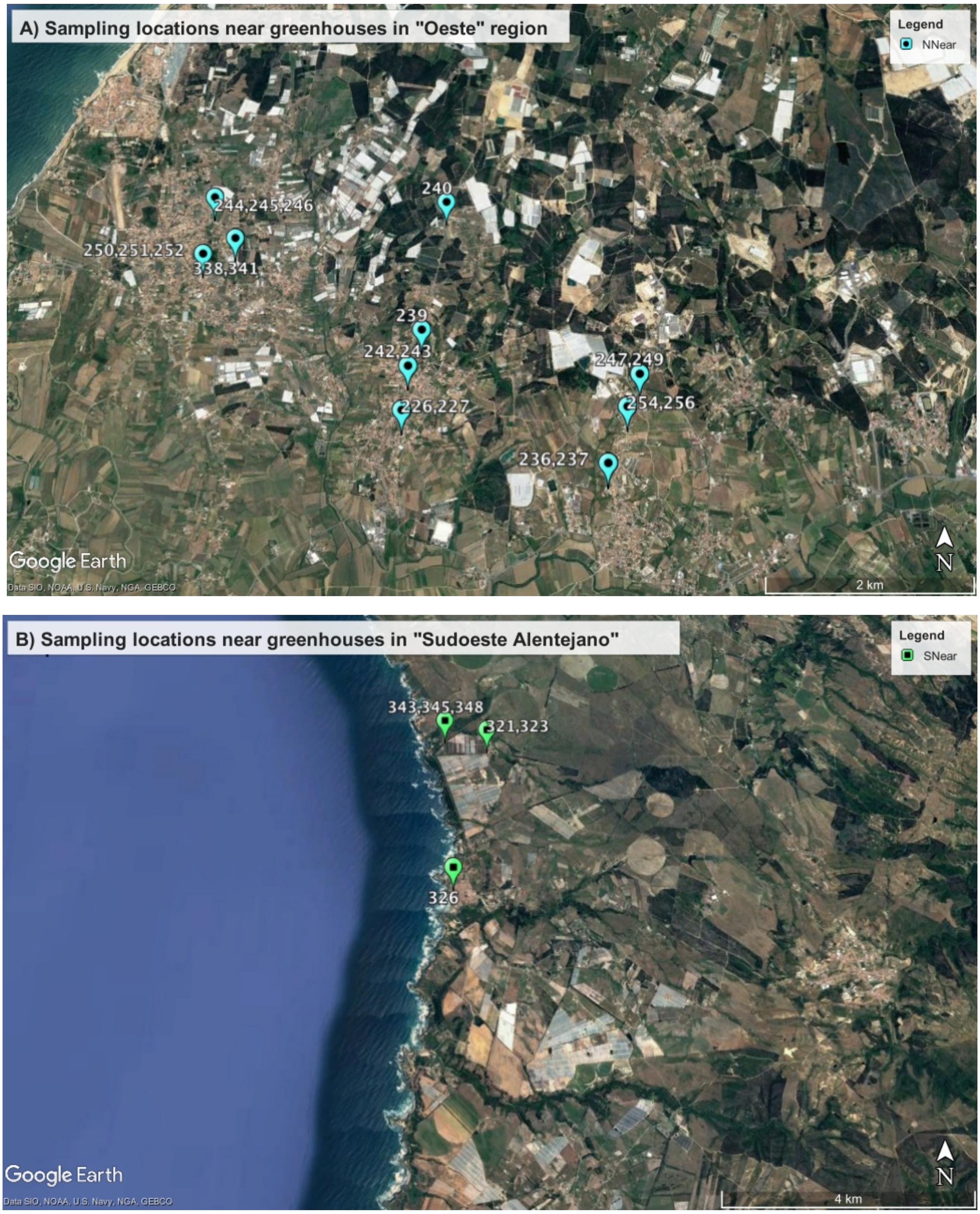

Supplementary Figure S1 – Sampling locations near greenhouses in "Oeste" (*NNear*) and "Sudoeste Alentejano" (*SNear*), Portugal (Map data: Google Earth). Numbers represent the identification codes of individuals bumblebees.

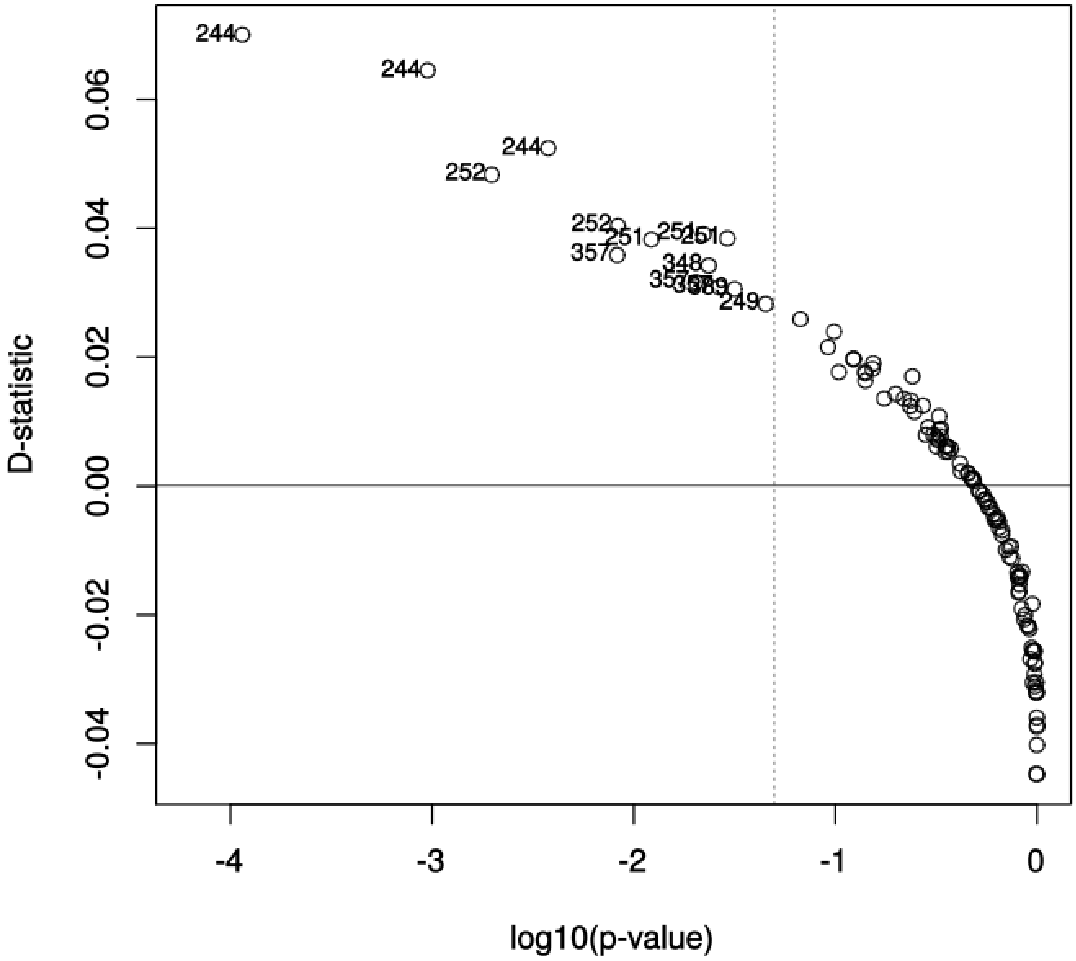

Supplementary Figure S2 – P-values (in log10 scale) of the D-statistic tests at the individual level, corresponding to the results in Fig. 4. One tailed test for positive D-statistic significantly different from zero. P-values obtained using a block-jackniffe approach. The label of individuals with significant positive D-statistic are shown next to the corresponding points.

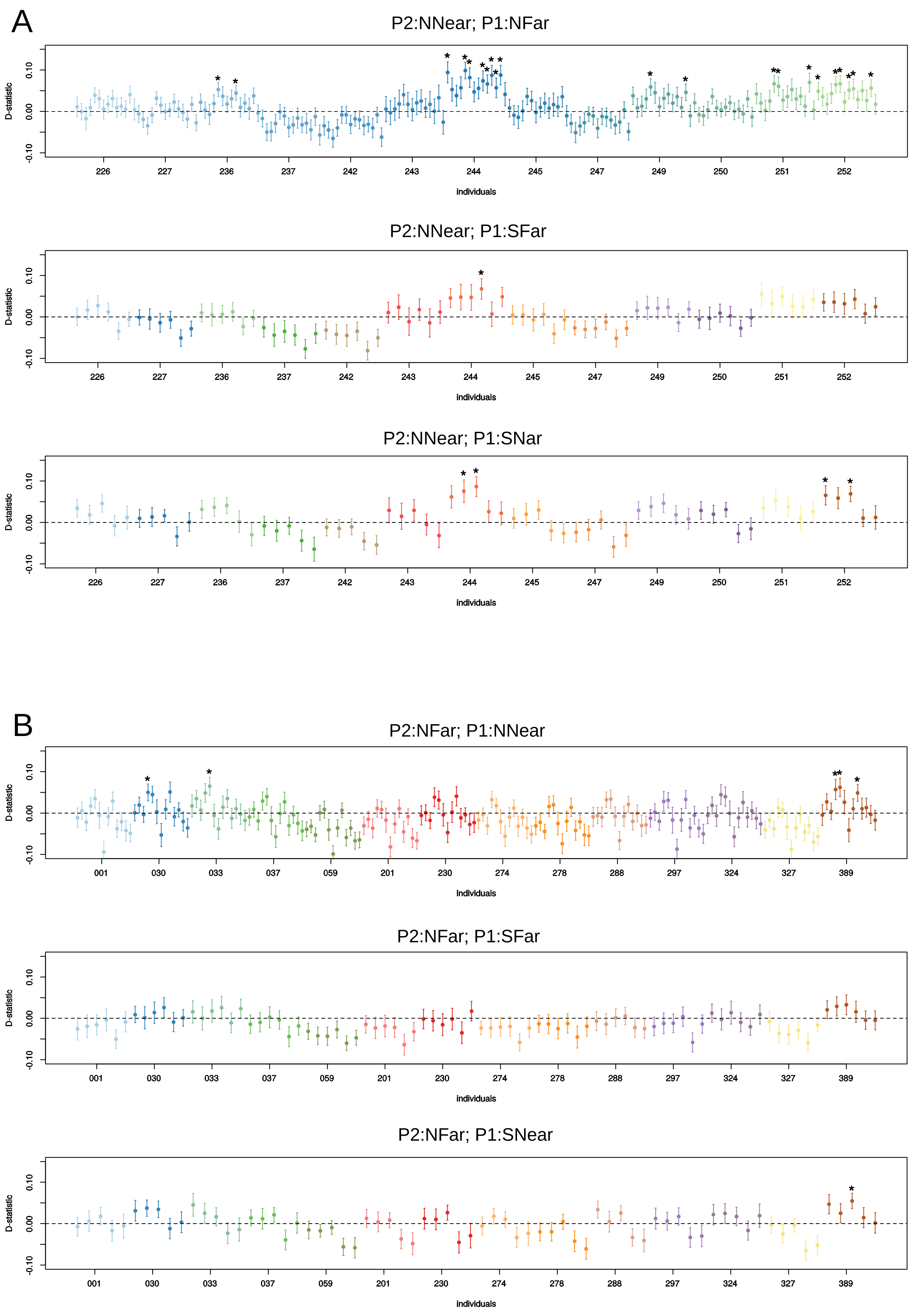
A
P2:NNear; P1:NFar
P2:NNear; P1:SFar
P2:NNear; P1:SNar
D-statistic
-0.10
0.00
0.10
226
227
236
237
242
243
244
245
247
249
250
251
252
individuals
B
P2:NFar; P1:NNear
P2:NFar; P1:SFar
P2:NFar; P1:SNear
001
030
033
037
059
201
230
274
278
288
297
324
327
389

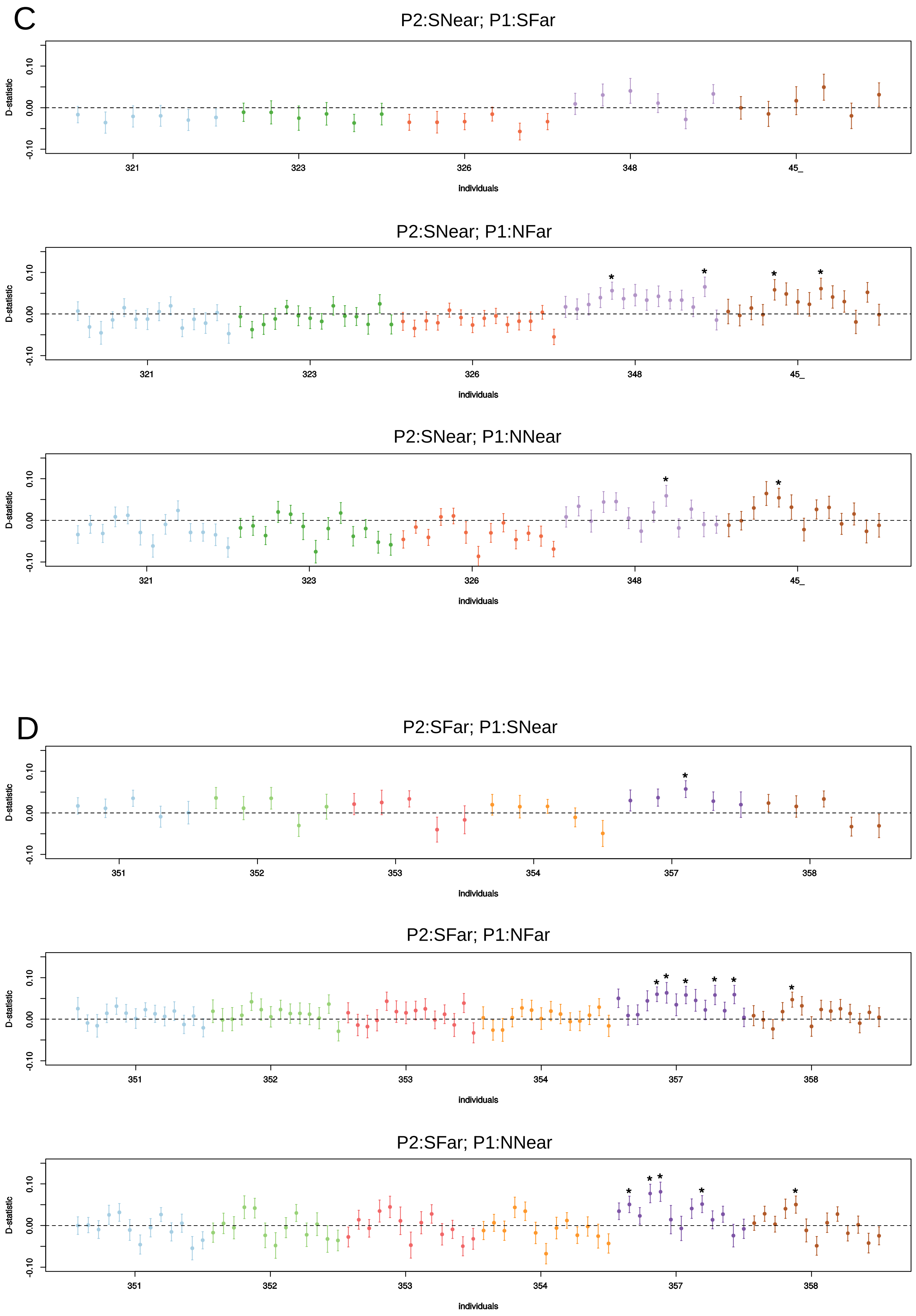
C
P2:SNear; P1:SFar
D-statistic
0.10
0.00
-0.10
321
323
326
348
45_
individuals
P2:SNear; P1:NFar
P2:SNear; P1:NNear
D
P2:SFar; P1:SNear
351
352
353
354
357
358
P2:SFar; P1:NFar
P2:SFar; P1:NNear

Supplementary Figure S3 – D-statistic results per individual in the target population, P2 (A – *NNear*, B – *NFar*; C – *SNear*, D - *SFar*) against each individual of population P1. We performed this test due to the heterogeneity of populations that could include individuals with past gene flow with Commercial in their recent ancestral history. Results are consistent with the ones in Fig. 4, indicating that the signature of gene flow with Commercial (*CH*) is robust. Asterisks indicate significant p-values ($p<0.01$).

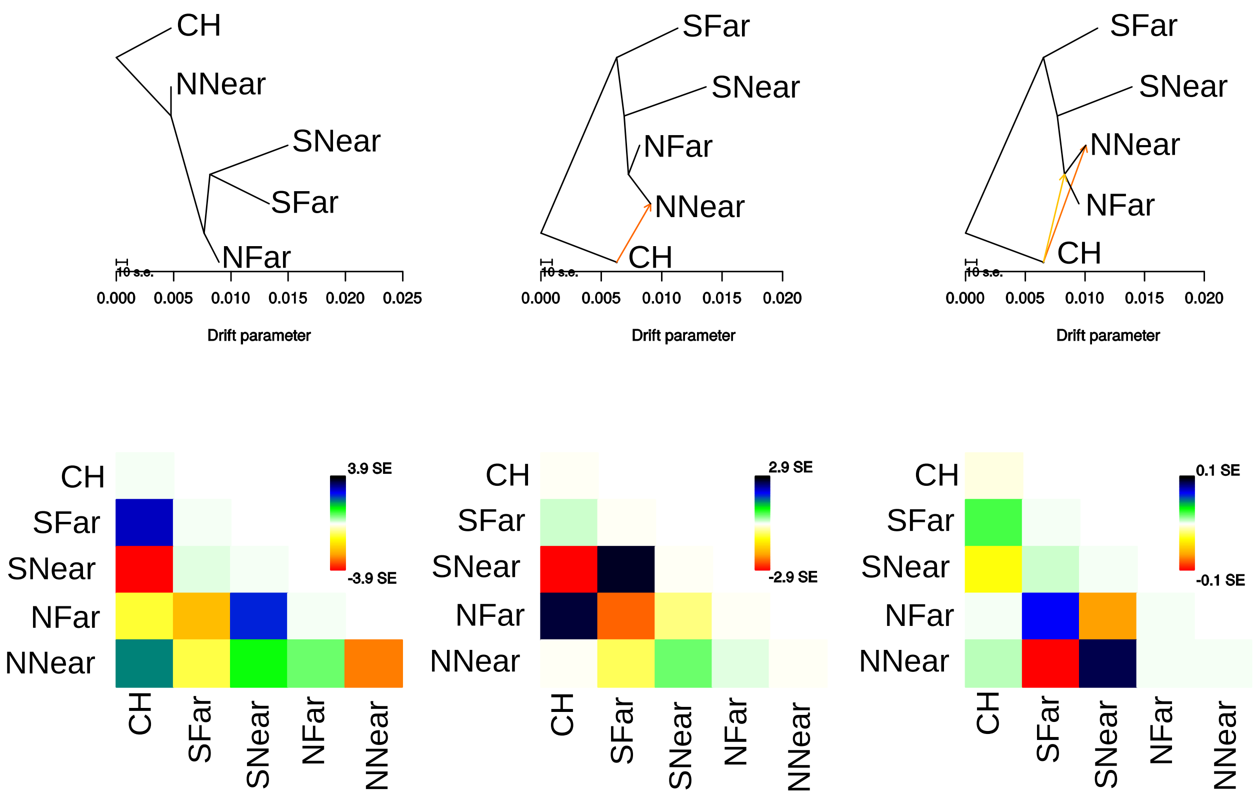

Supplementary Figure S4 – TreeMix results for different number of migration edges added to the model. (a-c) Inferred population tree and migration direction for models with 0, 1 and 2 migration edges. (d-f) fit of the covariance matrix for models with 0, 1, and 2 migration edges, shown as the normalized difference between the expected and observed covariance matrix (note the different scale for models with increasing migration edges). The fit of the expected to the observed co-variance matrix increases considerably when adding migration edges. The inferred migration edges indicate recent introgression from Commercial into NNear (for 1 and 2 migration edges).

Supplementary Table S1 – Individual information for: sampling (date of collection, location, sex); COI haplotype; RAD sequencing outputs (number of initial and mapped reads) mtDNA haplotype; analysis outputs (excel file)
